# Pathogenicity and phenotypic sulfadiazine resistance of*Toxoplasma gondii* isolates obtained from livestock in northeastern Brazil

**DOI:** 10.1590/0074-02760150459

**Published:** 2016-06

**Authors:** Claudio BS Oliveira, Ywlliane SR Meurer, Joelma MA Andrade, Maria ESM Costa, Milena MC Andrade, Letícia A Silva, Daniel CF Lanza, Ricardo WA Vítor, Valter F Andrade-Neto

**Affiliations:** 1 Universidade Federal do Rio Grande do Norte, Centro de Biociências, Departamento de Microbiologia e Parasitologia, Laboratório de Biologia da Malária e Toxoplasmose, Natal, RN, Brasil; 2 Universidade Federal do Rio Grande do Norte, Centro de Biociências, Departamento de Fisiologia, Laboratório de Estudos da Memória, Natal, RN, Brasil; 3 Universidade Federal de Minas Gerais, Instituto de Ciências Biológicas, Departamento de Parasitologia, Belo Horizonte, MG, Brasil; 4 Universidade Federal do Rio Grande do Norte, Centro de Biociências, Departamento de Bioquímica, Laboratório de Biologia Molecular Aplicada, Natal, RN, Brasil

**Keywords:** atypical profiles, pathogenicity, sulfadiazine-resistance, Toxoplasma gondii

## Abstract

*Toxoplasma gondii* is the causative protozoan agent of toxoplasmosis, which is a common infection that is widely distributed worldwide. Studies revealed stronger clonal strains in North America and Europe and genetic diversity in South American strains. Our study aimed to differentiate the pathogenicity and sulfadiazine resistance of three *T. gondii*isolates obtained from livestock intended for human consumption. The cytopathic effects of the *T. gondii* isolates were evaluated. The pathogenicity was determined by polymerase chain reaction-restriction fragment length polymorphism (PCR-RFLP) using a CS3 marker and in a rodent model in vivo. Phenotypic sulfadiazine resistance was measured using a kinetic curve of drug activity in Swiss mice. IgM and IgG were measured by ELISA, and the dihydropteroate synthase (DHPS) gene sequence was analysed. The cytopathic effects and the PCR-RFLP profiles from chickens indicated a different infection source. The Ck3 isolate displayed more cytopathic effects in vitro than the Ck2 and ME49 strains. Additionally, the Ck2 isolate induced a differential humoral immune response compared to ME49. The Ck3 and Pg1 isolates, but not the Ck2 isolate, showed sulfadiazine resistance in the sensitivity assay. We did not find any DHPS gene polymorphisms in the mouse samples. These atypical pathogenicity and sulfadiazine resistance profiles were not previously reported and served as a warning to local health authorities.

Toxoplasmosis is a zoonotic disease caused by the protozoan *Toxoplasma gondii*, which is an obligate intracellular parasite of warm-blooded animals, that affects one-third of the human population worldwide (Tenter et al. 2000)*.* This opportunistic human pathogen induces a devastating disease in immunocompromised individuals, especially HIV/AIDS patients and congenitally infected neonates (Montoya & Liesenfeld 2004), which requires strong medical care (Vaillant et al. 2005, Sukthana 2006). Toxoplasmosis is the third most common cause of hospitalisation due to food-borne infections in the United States (Mead et al. 1999). In Europe, the consumption of undercooked infected meat is responsible for 30-63% of *T. gondii* infections (Cook et al. 2000, Tenter et al. 2000).

Classically, *T. gondii* had been classified into three genetic clonal lineages (Types I, II, and III) (Howe & Sibley 1995). Recently, a new group of *T. gondii* strains referred to as haplogroup 12 was described in North America (Khan et al. 2011). Clonal propagation is likely favoured due to the ability of this parasite to be transmitted between intermediate hosts via the ingestion of tissue cysts, which is a trait that distinguishes it from related parasites (Su et al. 2003). *T. gondii*exhibits a large amount of diversity in Brazil and other South American countries (Carneiro et al. 2013), probably due to parasite sexual reproduction in several distinct feline species (Khan et al. 2011).

One study revealed that parasites obtained in the Rio Grande do Norte state exhibited atypical pathogenicity in rodent models (Andrade et al. 2013). Moreover, clinical studies showed that this state presented a high rate of vulnerable women of childbearing age (Barbosa et al. 2009) and atypical cases of ocular toxoplasmosis (Mendes et al. 2014). Thus, this study aimed to standardise the maintenance of atypical strains in vitro and in vivo and to ascertain the pathogenicity and phenotypic sulfadiazine resistance of three *T. gondii* isolates obtained from livestock intended for human consumption in the state of Rio Grande do Norte in northeastern Brazil.

## MATERIALS AND METHODS

*Cell lineages and animals* - The murine immortalised macrophage RAW 264.7 cell line (Sigma-Aldrich, St. Louis, MO, USA) was cultured in Dulbecco’s modified Eagle’s medium (DMEM; GIBCO Inc., NY, USA) supplemented with 40 mg/L of gentamicin and 10% foetal bovine serum (FBS; GIBCO Inc., NY, USA). The cells were incubated in an atmosphere of 5% CO_2_ at 37ºC and sub-cultured every seven days. This cell line is often used to represent part of the immune response that interacts directly with the parasite in mice (Andrade et al. 2006, Lang et al. 2006).

Female C57BL/6 inbred strain and Swiss Webster outbred mice (6-8 weeks old and 22-28 g of weight) were used for the pathogenicity and sulfadiazine resistance assays. The mice were housed and offered drinking water and regular mouse feed *ad libitum*.

The study was performed using the Guidelines for Ethical Conduct in The Care and Use of Animals from the Federal University of Rio Grande do Norte (CEUA, Protocol number 46/2013).

*Parasite isolation* - The *T. gondii* serological analysis was performed in blood, heart tissue, and brain tissue samples collected from 223 sheep, 50 goats, 18 pigs and 43 free-range chickens (Andrade et al. 2013). Seropositive tissues were selected and digested with pepsin at 37ºC for 90 min according to the method of Dubey et al. (2002). Three *T. gondii* isolates were obtained from the samples and maintained under stable growth conditions in both in vivo and in vitro systems. These three isolates represented a sampling of this group and were obtained from one pig and two chickens intended for human consumption. Thus, these isolates may represent a risk for human health and may be involved in the atypical clinical manifestations that occur in the region (Mendes et al. 2014).

*Parasites* - The *T. gondii* isolates used in this study were TgCkBrRN2 (Ck2) and TgCkBrRN3 (Ck3) from chickens and TgPgBrRN1 (Pg1) from a pig (Andrade et al. 2013). The farm animals were often used for human consumption in Rio Grande do Norte state.

The *T. gondii* clonal lineages used in this study were the strains of*T. gondii* RH, ME49 and VEG were kindly provided by Toxoplasmosis Laboratory, Department of Parasitology, ICB/UFMG. The RH strain (Type I) is highly pathogenic for all mouse lineages, and a minimal infective tachyzoite inoculum can be lethal for the intermediate hosts. The ME49 strain (Type II) is mildly virulent in murine models, whereas the VEG strain (Type III) is typically avirulent.

*Maintenance of the parasite isolates in vitro and in vivo* - For proliferation in vitro, 150 cysts were inoculated intraperitoneally into C57BL/6 mice (n = 3). After three days, all mice were euthanised, and tachyzoites were recovered from the peritoneal exudate. The obtained parasites were inoculated into RAW cell cultures and observed daily with an inverted microscope.

For in vivo maintenance, five cysts were inoculated *per gavage* from Swiss mice (n = 4). Three days after infection, the water provided to the animals was replaced with a 500 mg/L sulfadiazine solution (Dubey 2010) for 10 days. After this period, the animals were returned to pure water and then followed to monitor the chronicity of infection.

*Determination of cytopathic effects* - Raw 264.7 cells were seeded into 24-well microplates at a density of 1 × 10^5^ cells per well and incubated at 37ºC for 24 h. Then, the cells were infected at a ratio of two parasites/cell using the Ck2 or Ck3 isolate or a standard virulent Type I (RH) or avirulent Type II (ME49) clonal lineage. The infected cells were observed, and images were taken with a Zeiss Axiovert microscope (Carl Zeiss Inc., Thornwood, NY, USA) equipped with a camera plus a CO_2_ incubator system. For recordings, images were collected every 30 min for 48 h. The images were subjected to a qualitative analysis with the start time and the presence of cell lysis used as criteria for the classification of the cytopathic effects.

*Pathogenicity in vivo* - The pathogenicity of the isolates was determined in a mouse model (Dubey et al. 2002). Two infection routes were tested.

By the oral route: outbred Swiss Webster (n = 3) or inbred C57BL/6 (n = 3) mice were infected orally with five cysts obtained from mice previously infected with Ck2, Ck3, Pg1 or the standard clonal lineage ME49. This approach is widely used and has the ability to simulate the natural route of infection more accurately (Dubey et al. 1998, Alvarado-Esquivel et al. 2011, Wang et al. 2011).

By the intraperitoneal route: Swiss mice (n = 3) were infected with 5 x 10^4^ tachyzoites. The parasites were previously obtained from a RAW 264.7 cell culture infected with a clonal lineage (RH, ME49 or VEG) or local isolate (Ck2, Ck3 or Pg1). In both pathways, a cumulative mortality rate was obtained by following the animals for 30 days. Serum samples were collected from the surviving animals 0, 15 and 30 days post-infection by orbital puncture for confirmation of infection by ELISA. The analysis of the different infection routes aimed to determine whether clinically differential evolution occurred when infection was initiated by cysts or tachyzoites.

*Polymerase chain reaction-restriction fragment length polymorphism (PCR-RFLP) at the CS3 locus* - The target DNA sequence of the CS3 marker was ampliﬁed by PCR using internal primers as previously described (Silva et al. 2014). Because the DNA was extracted from purified tachyzoites, no previous amplification with the external primers was necessary. The amplification conditions were the same as those used for the multi-locus PCR-RFLP genotyping (Pena et al. 2008) except that 2.5 µL of template DNA was used and the annealing temperature of the cycles was 63ºC for 30 s. The PCR products were double digested with N1aIII and MboI (New England BioLabs) (Silva et al. 2014). The digested products were later purified by extraction with equal volumes of phenol:chloroform (1:1). The DNA banding pattern was revealed by staining 5% polyacrylamide gels with silver nitrate and photographed. The RH (type I), ME49 (type II), and VEG (type III) clonal strains were used as references. Additionally, recombinant strains (CTBR24 and CTBR21) carrying unusual alleles were included (Pena et al. 2008).

*Anti-T. gondii IgM and IgG ELISAs* - The serum IgG and IgM anti-*T. gondii* antibody concentrations were measured by enzyme-linked immunosorbent assay (ELISA) based on the protocol of Alvarado-Esquivel et al. (2011). First, 96-well flat-bottomed microtitre plates (Greiner Bio-One GmbH, Frickenhausen, Germany) were coated overnight at 4ºC with 100 µL of *T. gondii* lysate antigen (TLA) at a final concentration of 1 μg/mL in 50 mM sodium carbonate buffer (pH 9.6). The plates were washed four times with phosphate-buffered saline (PBS) containing 0.05% Tween 20 (PBS-T) (pH 7.4).

The plates were blocked with 200 µL of a 2% skim milk powder solution (Molico-Nestlé®, Araçatuba, SP, Brazil) for 1 h at 37ºC. Then, 1/200 dilutions of serum samples in PBS (200 μL/well) were added to the wells and incubated for 1 h at 37ºC. After washing the plates four times, one hundred microliters of rabbit anti-mouse IgG or IgM-HRP (Sigma-Aldrich, St. Louis, MO, USA) diluted 1:10,000 (Anti-IgG) or 1:1,000 (Anti-IgM) in PBS, respectively, was added to each well. After 1 h at 37ºC, the plates were washed five times with PBS-T. Thereafter, the plates were incubated for 10 min at room temperature with 50 μL/well of TMB (Invitrogen-Life Technologies Gaithersburg, MD, USA). The reaction was stopped by adding 30 μL/well of 4N H_2_SO_4_ and the absorbance was read at 450 nm.

*Evaluation of the sulfadiazine resistance phenotype in vivo* - Swiss mice (n = 4) were infected orally with five cysts obtained from animals infected for thirty days with Ck2, Ck3, Pg1 or the standard clonal lineage ME49. 24 h post-infection, the animals were administered sulfadiazine (100, 200 or 300 mg/kg) daily for six days based on the method of Oliveira et al. (2014). The mice were observed for 30 days after infection. Infection in the surviving mice was confirmed by the presence of brain cysts.

*Primer design and dihydropteroate synthase (DHPS) gene sequencing* - A 396 bp region of the DHPS coding sequence corresponding to positions 968 to 1364 (considering the first ATG of the coding region as the start site) was amplified from the total DNA of the Ck2, Ck3 and Pg1 strains using the primers DHPS407F (5’-ACGCGGATCAGATAATCAAGG-3’) and DHPS407R (5’-ACAGCATCTCTCGCGACT-3’). This region covers codon 407, which is associated with sulfadiazine resistance. The PCR was performed in a 20 µL reaction containing 0.25 µM of each primer, 1.5 mM MgCl_2_, 1U of Taq (Ludwig), 0.2 mM of each dNTP and 1X reaction buffer. The amplification was performed using a Life Touch Thermal Cycler TC-96 (BIOER) with the following cycle parameters: initial denaturation at 94ºC for 2 min, followed by 35 cycles of 94ºC for 40 s, 57ºC for 40 s and 70ºC for 40 s and a final extension at 70ºC for 3 min. Then, the PCR products were analysed in a 1.5% agarose gel stained with ethidium bromide. The amplicons were purified and sequenced using the Applied Biosystems® 3500 Genetic Analyzer platform according to the manufacturer’s specifications. All amplicons were sequenced at least twice (forward and reverse), and the electropherograms were analysed using BioEdit version 7.2.5.

*Statistical analysis* - The data were analysed with a multiple*T*-test or one-way analysis of variance (ANOVA). The GraphPad 5.0 software was used for graphical design (GraphPad Software, La Jolla, CA, USA) and statistical analysis. Differences were considered significant when p < 0.05.

## RESULTS

**Fig. 1:**
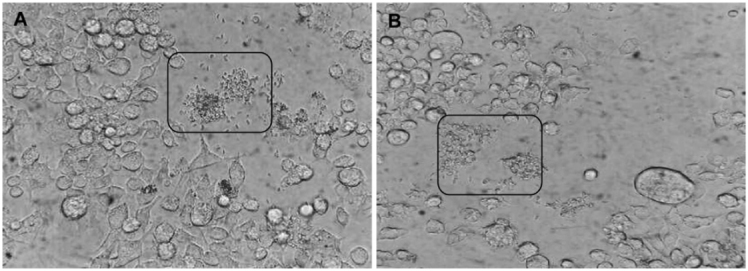
: RAW 264.7 cell cultures infected with Ck2 (A) or Ck3 (B). Frames and arrows highlight extracellular tachyzoites.

**Fig. 2:**
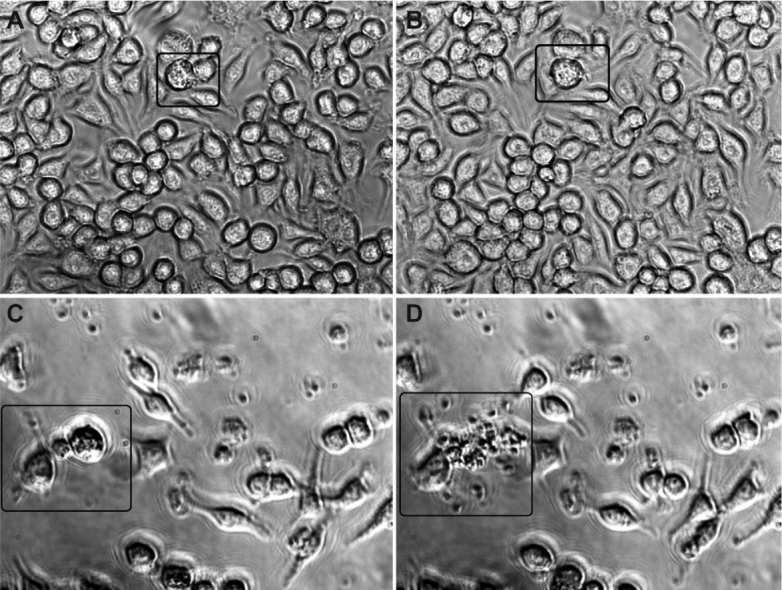
: RAW 264.7 cell cultures infected with Ck2 (A-B) or Ck3 (C-D). Images A-B were taken 17.5 h and 20 h post-infection. Images C-D were taken 22.5 h and 23 h post-infection.

*Maintenance of the parasite, pathogenicity in vivo and cytopathic effects* - There was no evidence of any major morphological variation among the three groups (RH, Ck2 and Ck3) in the RAW 264.7 cell cultures (Fig. 1). However, we observed faster cell lysis with isolate Ck3 compared to isolate Ck2 (Fig. 2).

No cell lysis was observed after 48 h of infection with isolate Ck2. All cells remained undamaged, even when they contained a large cytoplasmic parasite load.Fig. 2 shows a cell with a high parasitic load that retained its structure at 17.5 h (Fig. 2A) and 20 h (Fig. 2B) post-infection.

Fig. 2C-Dshows the patterns of cytopathic effects of isolate Ck3. Fig. 2C demonstrates a cell 22.5 h post-infection that harbours a large number of parasites but does not exhibit lysis. In Fig. 2D, lysis of the infected cell was observed 23 h post-infection, indicating the beginning of the period of cell death caused by this*T. gondii* isolate in RAW 264.7 cell cultures.

**Fig. 3:**
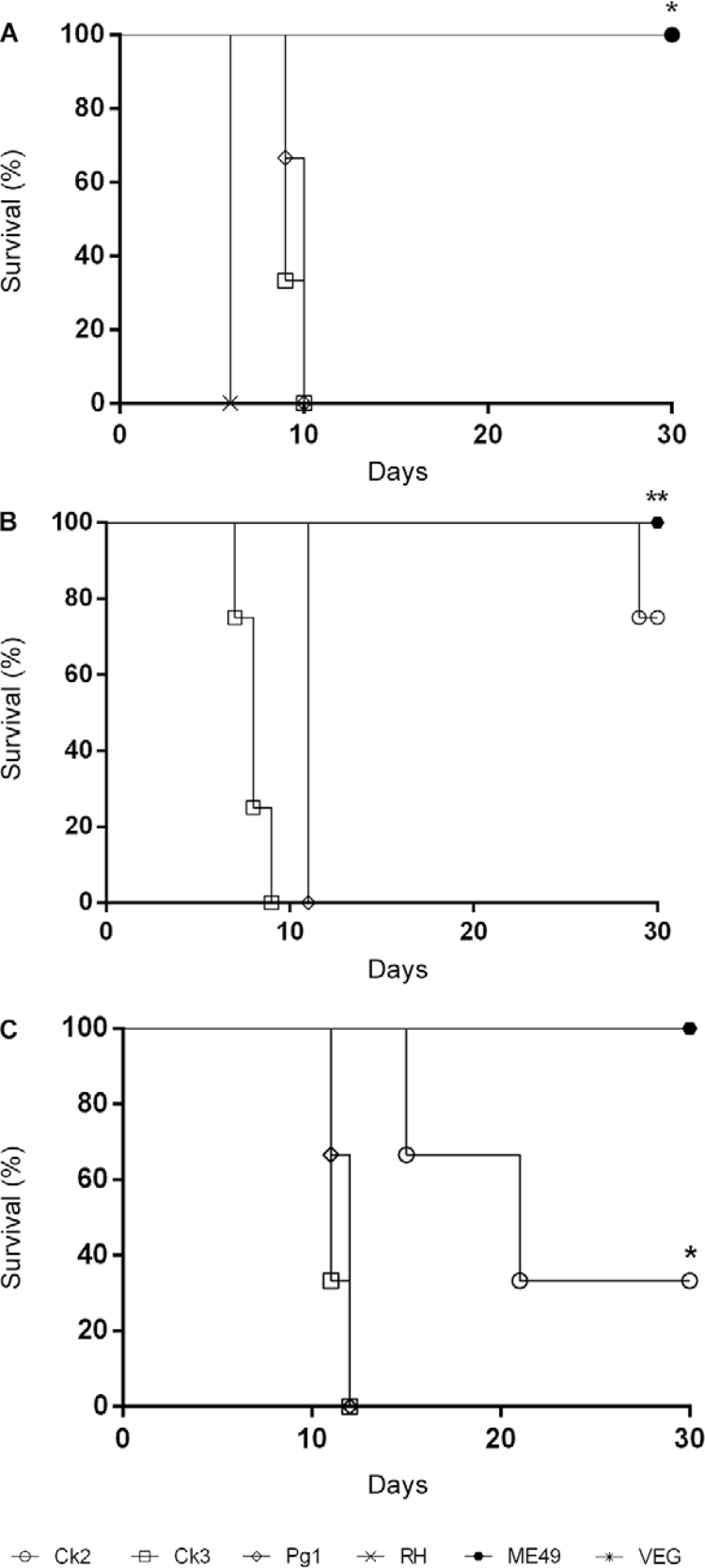
: survival curve for the assessment of pathogenicity. (A) Swiss female mice (n = 3) after infection with 5 x 104 Ck2, Ck3 or Pg1G*Toxoplasma gondii* tachyzoites or the RH, ME49 or VEG clonal lineage controls; (B) Swiss female mice (n = 3) after infection with five cysts of the Ck2, Ck3, Pg1 or ME49 strain; (C) C57BL/6 female mice (n = 3) after infection with five cysts of the Ck2, Ck3, Pg1 or ME49 strain (***p < 0.001; **p < 0.01; *p < 0.05).

Fig. 3A-Brevealed that the pathogenicity in mice was similar for the different routes of infection tested in this study (oral or intraperitoneal). All clonal strains used as controls exhibited typical mortality patterns. This same pattern was repeated in inbred C57BL/6 mice. We also observed a distinct pattern for the Ck2 isolate (Fig. 3C) that was similar to the pattern observed in the Swiss mice.

*PCR-RFLP at the CS3 locus* - Restriction fragment length polymorphisms at the CS3 locus of these three isolates showed that the Ck3 isolate harboured allele I, the Ck2 isolate harboured allele III and the Pg1 isolate harboured allele II.

**Fig. 4:**
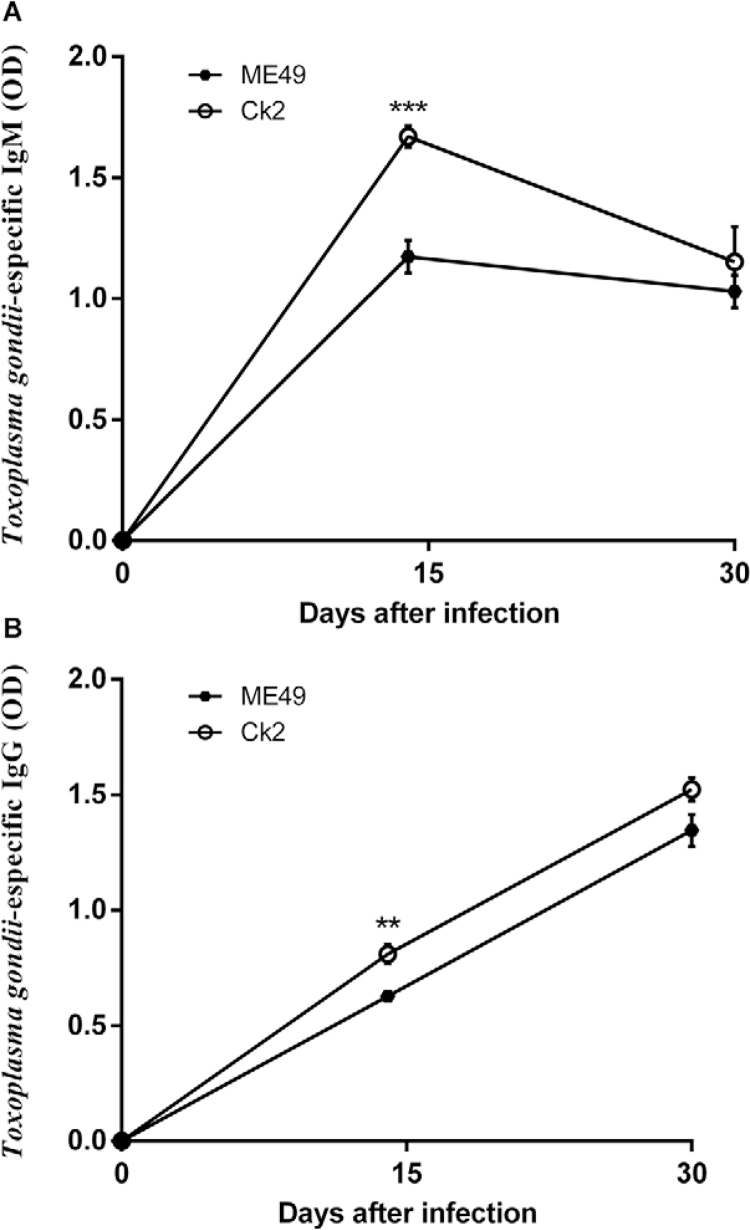
: serum IgM (A) and IgG (B) levels in C57BL/6 female mice (n = 3) infected with five cysts of the Ck2 or ME49 strain (***p < 0.001; **p < 0.01; *p < 0.05).

*Immunoglobulin dosage* - This experiment allowed us to quantify the acute phase (IgM) and chronic phase immunoglobulins (IgG) in C57BL/6 infected with Ck2, Ck3, Pg1 or ME49. Animals infected with Ck2 or ME49 *T. gondii*survived until the last blood collection; thus, only these groups were selected for this analysis. There was a significant increase in specific anti-*T. gondii* IgG (Fig. 4A) and IgM (Fig. 4B) in the Ck2 group 15 days post-infection. No significant changes in antibody production were observed thirty days post-infection. The IgM and IgG curves for the ME49 strain followed a characteristic pattern.

**Fig. 5:**
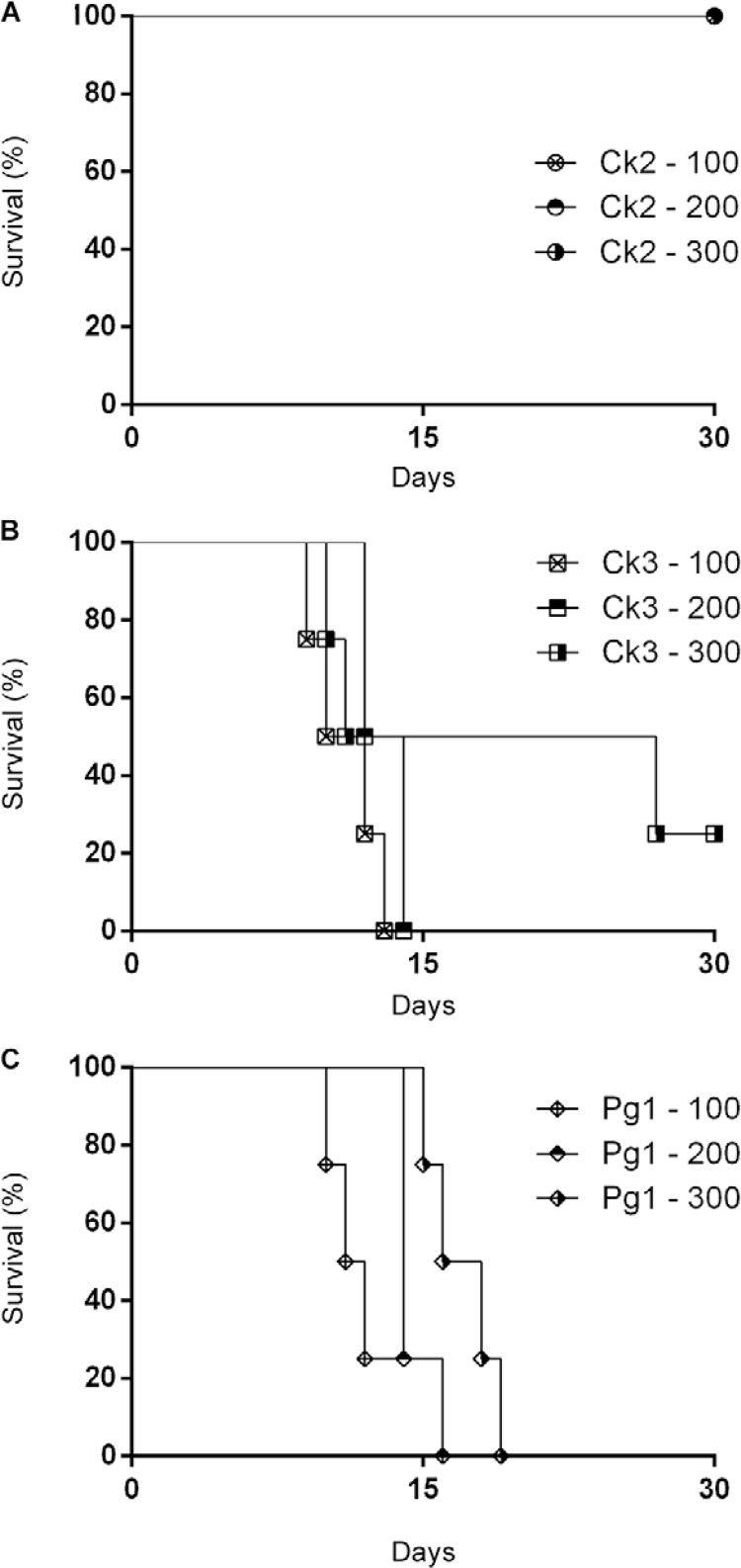
: percent survival of Swiss mice infected with five cysts of the Ck2 (A), Ck3 (B) or Pg1 (C) isolates of *Toxoplasma gondii*and treated with different concentrations of sulfadiazine (100, 200 or 300 mg/kg/day) for six days.

*Phenotypic sulfadiazine resistance in vivo* - An analysis of the survival curves of infected animals treated with 100, 200 or 300 mg/kg of sulfadiazine presented different profiles (Fig. 5). Isolate Ck2 and the ME49 strain clone showed high sensitivity to sulfadiazine because it was possible to calculate an overall survival rate for both (Fig. 5A). In contrast, Ck3 and Pg1 showed a profile of resistance to sulfadiazine because animals infected with Ck3 and treated with sulfadiazine had high mortality rates (Fig. 5B) and animals infected with Pg1 presented 100% mortality in all treated groups (Fig. 5C). No mortality was observed in the animals infected with the ME49 strain and treated with sulfadiazine.

Sequencing of the sulfadiazine resistance-associated DHPS gene from the Ck2, Ck3 and Pg1 parasites showed no polymorphisms (Supplementary figure).

## DISCUSSION

The consumption of raw or undercooked meat dishes is a remarkable infection route for*T. gondii* (Sukthana 2006) because manifold viable cysts remain in host tissues (Tenter et al. 2000, Kijlstra & Jongert 2008, Halos et al. 2010). In the previous report, Andrade et al. (2013) showed the pathogenicity profiles of atypical *T. gondii* strains from farm animals used for human intake in murine models. However, the authors failed to evaluate the infection routes or normalise the therapeutic response against the atypical strains; these approaches can help explain the atypical archetypes of the *T. gondii* strains (Mendes et al. 2014).

Recently, Su et al. (2010) proposed an integrated molecular approach for the detection and identification of *T. gondii* based on a variety of genetic markers, including SAG1, SAG2 (3’SAG2 and 5’SAG2), SAG3, BtuB, GRA6, c22-8, c29-2, L358, PK1, new SAG2, and APICO. These related approaches can distinguish isolates and discriminate between different degrees of strain pathogenicity, although they are only effective with typical clonal lineages such as those that occur in the United States (Khan et al. 2011) and Europe (Howe & Sibley 1995). The atypical strains reported in several countries in South America including Brazil (Pena et al. 2008) show another clonal strain profile that is sometimes absent (Ferreira et al. 2006, Khan et al. 2006) and poorly detectable with the available molecular tools (Dubey et al. 2008). Indeed, the current markers are not useful for the detection of*T. gondii* strains with large polymorphisms, such as those obtained in Brazil. Thus, molecular tools need to be developed for this approach.

In this study, we cultivated atypical strains in vitro and in vivo using an experimental method for future evaluations with molecular and phylogenetic approaches. Our analysis revealed differential in vitro cytopathic effects of the Ck2 and Ck3 isolates. The results showed that Ck2 did not induce the death of infected cells 48 h post-infection, whereas the Ck3 isolate caused cell death 23 h post-infection. Here, we proposed a new marker to complement and enhance the CS3 model proposed by Su et al. (2010), which is proving to be effective in discriminating the pathogenicity of atypical strains of the parasite. The use of this marker enables the pathogenicity to be more clearly differentiated. Isolate Ck2, which harbours allele III at the CS3 locus, presented low pathogenicity in mice, whereas isolates Ck3 and Pg1, which harbour alleles I and II, respectively, presented higher pathogenicity. These results confirm the utility of the CS3 marker for predictions of the pathogenicity of Brazilian *T. gondii* isolates in mice and are consistent with earlier studies showing that alleles I and II are associated with mortality of infected mice, whereas allele III is associated with survival (Pena et al. 2008, Ragozo et al. 2010, Silva et al. 2014).

We performed an in vivo pathogenicity analysis with a standardised inoculum and two infection routes (oral and intraperitoneal). This analysis confirmed the different infectivity profiles of the isolates. The Ck3 isolate was the most pathogenic, followed by Pg1 and Ck2. According to Carneiro et al. (2013), the Pg1 and Ck3 isolates can be classified as pathogenic, whereas Ck2 presents as an intermediate pathogenic isolate because it does not cause total mortality of the animals. We observed different pathogenicity profiles even for isolates obtained from the same region, suggesting that different *T. gondii* archetypal strains are present in the animals. Similarly, the infection route did not appear to interfere with the pathogenicity profiles of the strains. Thus, further evaluations should be performed using the oral route, which is similar to the natural course of infection (Dubey et al. 1998) and makes this route the preferred infection model. Only the Ck2 isolate showed survival 30 days post-infection. Therefore, we assayed IgM and IgG antibody production only with mice infected with this isolate. We observed significantly higher values for both antibodies 15 days post-infection compared to the ME49 strain’s antibody patterns. This finding can be explained by antigenic differences between the sampled *T. gondii* isolates.

Based on these results, toxoplasmosis treatment may cause a reduction in antibody production (Alvarado-Esquivel et al. 2011). Many reports have shown therapeutic failures against *T. gondii* by the standard drug sulfadiazine in rodent models (Alves & Vitor 2005, Alvarado-Esquivel et al. 2011). In this context, we investigated the resistance of each isolate to sulfadiazine (Kaye 2011). The Ck2-infected mice entered a chronic state with improved overall survival of the infected-treated animals. Conversely, the Pg1 or Ck3 infected-treated animals presented a partial or total mortality rate. We believe that the sulfadiazine resistance approach remains valid and has great value because molecular markers are not a reliable method to detect all *T. gondii* strain phenotypes (Doliwa et al. 2013a).

Although the sulfadiazine resistance corroborated with previous studies (Aspinall et al. 2002, Alves & Vitor 2005, Meneceur et al. 2008), the pattern of resistance presented in this study represented the first report of sulfadiazine resistance in atypical Brazilian*T. gondii* isolates in a natural infection rodent model. Alves and Vitor (2005) described a phenotypic profile of sulfadiazine resistance for *T. gondii* isolates in another infection model and demonstrated the frequency of natural resistance of Brazilian isolates, which may explain the therapeutic failure in 10% of patients with toxoplasmic encephalitis treated with sulfadiazine (Aspinall et al. 2002).

We sequenced the *T. gondii* DHPS gene from the isolates to evaluate polymorphisms associated with the phenotypic resistance profile to sulfadiazine because a point mutation might be involved in the mechanism of resistance. Few mutations associated with sulfadiazine resistance have been described to date. The most common mutation occurs in the DHPS sequence at the first nucleotide of codon 407, resulting in the replacement of Asn with Asp (Aspinall et al. 2002, 2012, Meneceur et al. 2008, Boughattas et al. 2011, Doliwa et al. 2013a). This codon is equivalent to codon 437 of the DHPS from*Plasmodium*, which is functionally linked to sulfadiazine resistance (Doliwa et al. 2013a). The resistant phenotypes may be associated with indirect mechanisms (Doliwa et al. 2013b). However, we did not find any amino acid changes in the DHPS gene region around nt 396, which comprises codon 407 of the DHPS sequence, in any of the analysed variants.

The absence of effective genetic markers is a limiting factor in research involving*T. gondii* strains with a phenotypic sulfadiazine resistance profile. In this context, new tools and proteomic analysis of the parasite are currently being applied. This method allowed the efficient evaluation of resistance to drugs in *Trypanosoma cruzi* (Andrade et al. 2008) and *Leishmania* spp. (Kumar et al. 2010). Previously, Doliwa et al. (2013b) demonstrated differences in the electrophoresis patterns of proteins involved in several different mechanisms, such as carbohydrate metabolism, host cell interaction, and protein translation, which were differentially expressed in sensitive or resistant*T. gondii* strains. Thus, although of the use of these proteins allows us to identify indirect resistance mechanisms to sulfadiazine, further studies are needed to validate potential new protein targets and improve current methodologies.

The emergence of *T. gondii* strains resistant to current drugs such as those described in this study represents a concern not only for treatment failure but also for increased clinical severity in reactivated infected or immunocompromised patients. For instance, in a previous report we related the clinical severity of retinochoroiditis lesions in ocular toxoplasmosis patients in the northeast region of Brazil (Mendes et al. 2014).

To summarise, we demonstrated that the Ck2 and Ck3 isolates were different phenotypic strains of *T. gondii*. The Ck3 and Pg1 isolates demonstrated greater pathogenic profiles and Ck2 presented an intermediate pathogenic pattern in a rodent model. Moreover, the infection route did not alter the pathogenicity patterns in the animals in this study. The currently accepted categorisation model for *T. gondii* is unsuitable for atypical strains. Therefore, we suggest the addition of the CS3 marker to aid in the detection and identification of non-archetypical isolates. Although we observed a distinct immune response triggered by the Ck2 strain, more analyses are needed. Finally, this was the first report in Brazil of a sulfadiazine resistance profile of *T. gondii* in a natural model of infection.
